# Fibrocystin in MDCK cells: impact on cell adhesion and epithelial morphogenesis

**DOI:** 10.1186/2194-7791-2-S1-A26

**Published:** 2015-07-01

**Authors:** Birga Sötje, Dieter Haffner, Wolfgang H Ziegler

**Affiliations:** Department of Pediatric Kidney, Liver and Metabolic Diseases, Hannover Medical School, Hannover, Germany

## Meeting abstract

Fibrocystin (FPC), a type I membrane protein of largely unknown function, is encoded by the *Pkhd1* gene, mutations of which cause autosomal recessive polycystic kidney disease (ARPKD). Among other potential functions, FPC appears to affect adhesion signaling of cells involving the FAK/Src axis. Contributions of epithelial cell adhesion and contractility to the disease process of ARPKD remain to be defined. The aim of this study is to elucidate the link between FPC function and the capacity of renal collecting duct epithelial cells to generate normal epithelia.

We analyze FPC function in MDCK II renal collecting duct epithelial cells applying 3D culture conditions and micro-pattered surfaces that confine adhesion of cells to induce formation of spheroids. In this setup, establishment of polarity and lumen depend on the capacity of cells to properly control cell adhesion and actomyosin contractility. We address the impact of altered cell behavior using modulators of cell contractility and adhesion signaling.

*Pkhd1* siRNA-treated MDCK cells and controls were employed to analyze spheroid formation, using specific markers for polarity, cell junctions and cilia. *Pkhd1* silencing strongly affects polarity and lumen formation in MDCK spheroids confirming involvement of FPC protein in cellular control of adhesion signals and establishment of polarity. Quantitative analysis of single cell adhesion reveals significant reduction in cell area and contact numbers. During spheroid formation, transient inhibition of actomyosin contractility by blebbistatin can restore epithelial morphogenesis of FPC-deficient MDCK cells.

In conclusion, loss of FPC disturbs adhesion signaling, cell-cell interaction, and control of cell tension resulting in impaired epithelial morphogenesis, which can be rescued by transient reduction of cell contractility. Using a cell-based model system, we can address molecular consequences and analyze rescue strategies of FPC deficiency in collecting duct epithelia.

